# The draft genome of horseshoe crab *Tachypleus tridentatus* reveals its evolutionary scenario and well-developed innate immunity

**DOI:** 10.1186/s12864-020-6488-1

**Published:** 2020-02-10

**Authors:** Yan Zhou, Yuan Liang, Qing Yan, Liang Zhang, Dianbao Chen, Lingwei Ruan, Yuan Kong, Hong Shi, Mingliang Chen, Jianming Chen

**Affiliations:** 1 State Key Laboratory of Genetic Engineering, School of Life Sciences, Fudan University, Shanghai, 200438 China; 2 Shanghai-MOST Key Laboratory of Health and Disease Genomics, Chinese National Human Genome Center at Shanghai, Shanghai, 201203 China; 3 Institute of Oceanography, Minjiang University, Fuzhou, 350108 China; 4 State Key Laboratory Breeding Base of Marine Genetic Resources, Fujian Collaborative Innovation Center for Exploitation and Utilization of Marine Biological Resources, Third Institute of Oceanography, Ministry of Natural Resources, 184 University Road, Xiamen, 361005 China; 5 Sino-French Hoffmann Institute, School of Basic Medical Sciences, Guangzhou Medical University, Guangzhou, 511436 China

**Keywords:** *Tachypleus tridentatus*, Genome, Evolution, Innate immunity, Coagulation

## Abstract

**Background:**

Horseshoe crabs are ancient marine arthropods with a long evolutionary history extending back approximately 450 million years, which may benefit from their innate immune systems. However, the genetic mechanisms underlying their abilities of distinguishing and defending against invading microbes are still unclear.

**Results:**

Here, we describe the 2.06 Gbp genome assembly of *Tachypleus tridentatus* with 24,222 predicted protein-coding genes. Comparative genomics shows that *T. tridentatus* and the Atlantic horseshoe crab *Limulus polyphemus* have the most orthologues shared among two species, including genes involved in the immune-related JAK-STAT signalling pathway. Divergence time dating results show that the last common ancestor of Asian horseshoe crabs (including *T. tridentatus* and *C. rotundicauda*) and *L. polyphemus* appeared approximately 130 Mya (121–141), and the split of the two Asian horseshoe crabs was dated to approximately 63 Mya (57–69). Hox gene analysis suggests two clusters in both horseshoe crab assemblies. Surprisingly, selective analysis of immune-related gene families revealed the high expansion of conserved pattern recognition receptors. Genes involved in the IMD and JAK-STAT signal transduction pathways also exhibited a certain degree of expansion in both genomes. Intact coagulation cascade-related genes were present in the *T. tridentatus* genome with a higher number of coagulation factor genes. Moreover, most reported antibacterial peptides have been identified in *T. tridentatus* with their potentially effective antimicrobial sites.

**Conclusions:**

The draft genome of *T. tridentatus* would provide important evidence for further clarifying the taxonomy and evolutionary relationship of Chelicerata. The expansion of conserved immune signalling pathway genes, coagulation factors and intact antimicrobial peptides in *T. tridentatus* constitutes its robust and effective innate immunity for self-defence in marine environments with an enormous number of invading pathogens and may affect the quality of the adaptive properties with regard to complicated marine environments.

## Background

Horseshoe crabs are marine arthropods, representing an ancient family with an evolutionary history record extending back approximately 450 million years [1]. Based on their static morphology and their position in the arthropod family tree, they have been therefore labelled “living fossils” for a long time [2]. There are now few types of existing horseshoe crabs with narrow distribution.

*Tachypleus tridentatus* (Leach, 1819), an extant horseshoe crab species, is mainly distributed from coastal Southeast China to western Japan and in a few islands in Southeast Asia [3]. Similar to other invertebrates, *T. tridentatus* relies entirely on its innate immune system, including haemolymph coagulation, phenoloxidase activation, cell agglutination, release of antibacterial substances, active oxygen formation and phagocytosis [4–8], which operates on pattern-recognition receptors (PRRs) upon the detection of pathogen-associated molecular patterns (PAMPs) present on surface of microbes, such as lipopolysaccharides, lipoproteins and mannans [9]. Upon recognition, PRRs trigger diverse signal transduction pathways, including the Toll pathway, IMD pathway, JAK-STAT and JNK pathways, that can produce immune-related effectors [10]. Previous studies have investigated important signalling pathways and gene families from other arthropods, such as insects, crustaceans and myriapods, revealing extensive conservation and functional diversity among innate immune components across arthropods [11, 12]. Currently, the immune molecular mechanisms of how horseshoe crabs achieve distinguishing “self” and “non-self” antigenic epitopes, also known as pathogen-associated molecular patterns (PAMPs), has not yet been established.

The Atlantic horseshoe crab, *Limulus polyphemus* (Linnaeus 1758), is the most extensively investigated species of horseshoe crabs, occupying a large latitudinal range of coastal and estuarine habitats along the west Atlantic coast from Maine to Florida in eastern North America and along the eastern Gulf and around the Yucatán peninsula of Mexico [3, 13, 14]. A previous research about the genome of *L. polyphemus* with a high assembly quality has published, focusing on the full repertoire of Limulus opsins, which could provide insight into the visual system of horseshoe crabs [15]. In order to obtain the genome characteristics not only of *T. tridentatus* but also of the xiphosuran lineage and try to reduce errors of only using a single draft-quality genome, the comparative genomic study of immune systems within *T. tridentatus* and *L. polyphemus* were included.

Here, we present an analysis of the *T. tridentatus* genome sequence together with comparative genomic and divergence time analyses on other available Chelicerata genomes to date, including the previously released *L. polyphemus* assembly [15]. Particular attention was paid to gene families related to assessing the genomic and phenotypic changes of horseshoe crabs, as well as exploring immune signalling pathways, antimicrobial peptides and coagulation factors that may contribute to their robust and effective innate immunity for self-defence in marine environments with enormous number of invading pathogens and may have important implications for the continuation of this species.

## Results

### General genome features

The genomic DNA isolated from *T. tridentatus* was sequenced to 124× coverage and assembled into a 2.06-Gb genome. The k-mer analysis yielded an estimated genome size of 2.22 Gb with a depth peak of 78×. The final draft assembly consists of 143,932 scaffolds with an N50 scaffold size of 165 kb, among which the longest scaffold size is 5.28 Mb and the shortest is 1 kb. The GC content of the genome is 32.03% (Table 1). A total of 24,222 protein-coding genes were conservatively predicted in the *T. tridentatus* genome in this study. The average exon and intron lengths predicted for the assembly are 333 bp and 3792 bp, respectively. A total of 88.25% of the predicted genes were assigned and annotated by comparing to the NCBI non-redundant database, KEGG database [16] and InterPro database [17].

**Table 1:** Summary of the *Tachypleus tridentatus* genome assembly and annotation statistic

Summary of the *Tachypleus tridentatus* genome assembly and annotation statistics.	
*Tachypleus tridentatus* assembly statistics	
Assembly size (Gb)	2.06
Number of scaffolds	143,932
N50 scaffold length (kb)	165
Largest scaffold (kb)	5278
Shortest scaffold (kb)	1
GC content	32.03%
Average exon length (bp)	333
Average intron length (bp)	3792
*Tachypleus tridentatus* assembly annotation statistics	
Total number of genes	24,222
% BUSCOs^a^	87.4 [10.8], 11.3, 1.3

^a^ of 1066 arthropod BUSCOs Complete [Duplicated], Fragmented, Missing, in the assembly

### Repeat annotation

The screening of repeat contents from the RepeatMasker [18] analysis based on similarity alignments identified 20.29 Mb in *T. tridentatus*, representing 0.99% of the genome size. Most of the identified repeat sequences were simple repeats (0.77%). To estimate of repeat sequences which are more difficult to detect in the draft assembly, RepeatModeler [19] was used to predict potential existing but unidentified repeats. Based on this analysis, repeat elements totalled 34.83% in *T. tridentatus*, including a 13.26% proportion of transposable elements. Meanwhile, long interspersed elements (LINEs) composed the largest portion at 6.21%. LTR elements (1.72%) and DNA elements (5.33%) were also detected in the *T. tridentatus* genome. To determine the reliability of the repeat contents screening by RepeatMasker and RepeatModeler, we also performed repeat analysis of the *L. polyphemus* genome for reference. Similar results were obtained with the identification of repeat sequences representing 1.11 and 34.24% in *L. polyphemus*, respectively. Given that RepeatMasker use similarity of known repeat sequences in the Repbase database to identify repeats in the input sequence, this suggests that the repeat sequences from horseshoe crabs have a great difference compared with existing homologous repeats.

### Assembly assessment

The completeness of the *T. tridentatus* genome assembly was assessed using the transcriptome data of the embryonic sample at Stage 21 (the hatch-out stage) of *T. tridentatus* [20]. It was found that 99.04% of the transcriptome contigs were aligned to the assembly scaffolds, with an e-value cut-off of 10^− 30^. To further confirm the completeness of the predicted genes, the commonly used genome assembly validation pipeline BUSCO [21] gene mapping method with 1066 BUSCO Arthropoda gene sets were utilized. The predicted genes of *T. tridentatus* reveals 98.7% conserved proteins of homologous species with 1052 BUSCOs (76.6% complete single-copy BUSCOs, 10.8% complete duplicated BUSCOs and 11.3% fragmented BUSCOs). Only 1.3% of the benchmarked universal single-copy orthologous groups of arthropod genes were missing in the assembly. This demonstrated that most of the evolutionarily conserved core genes were found in *T. tridentatus* genome, suggesting a remarkable completeness of genome assembly and predicted gene repertoire of *T. tridentatus*.

### Phylogeny analysis and divergence time dating

**Fig. 1:**
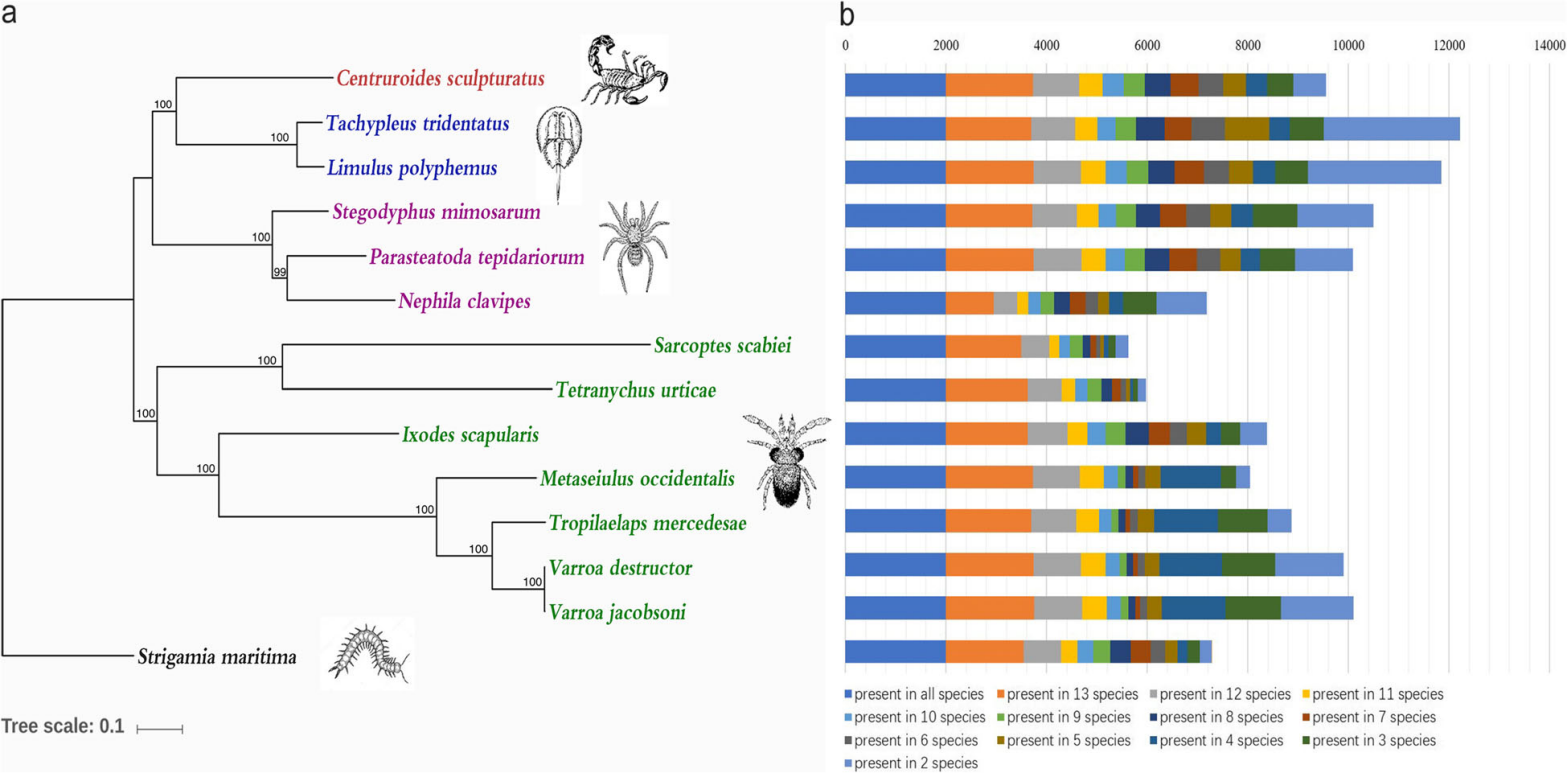
Comparative genomics. **a** Phylogenetic placement among *T. tridentatus* and other Chelicerata species. The phylogeny with 111 single-copy orthologous genes presented in all 14 species was built using RAxML. The tree was rooted with *S. maritima*. **b** Orthology comparsion among *T. tridentatus* and other Chelicerata species. There were 2720 (22.2%) and 2648 (21.5%) orthologs of *T. tridentatus* and *L. polyphemus* uniquely shared by the two species (major part of the corresponding light blue bar). *C. sculpturatus* had the most expanded species unique genes (7328), followed with 6247 *N. clavipes* specific genes. The number of species specific genes of *T. tridentatus* and *L. polyphemus* were in between with 1124 and 857, respectively. The images depicted in Figure 1 were redrawn by the authors according to picture source materials searched from Google images

Two *L. polyphemus* assemblies have been previously documented [15, 22], one of which was selected to perform comparative genomics according to a relatively higher assembly level. The OrthMCL [23] calculation resulted in a total of 12,116 orthologous groups in the genomes of *T. tridentatus* and *L. polyphemus*. Of these, 10,968 orthologues contained genes found in both horseshoe crab genomes, with 15,905 *T. tridentatus* and 20,390 *L. polyphemus* genes included; moreover, approximately 6880 of the shared genes were single-copy. Functional enrichment analysis showed that these shared genes were involved in several important pathways (*p*-value < 0.05), such as metabolic pathways (pyruvate, glycerolipid, amino sugar, nucleotide sugar and so on), ribosome biogenesis and DNA replication. The analysis also identified 1418 protein-coding genes that were only present in *T. tridentatus*. In total, 1956 genes were specific to *L. polyphemus*. To place *T. tridentatus* with the most current understanding of the evolution of Chelicerata species, phylogenetic and comparative genomic analyses of *T. tridentatus* and 11 other Chelicerata as well as one Myriapoda outgroup were conducted. The phylogenetic tree was rooted using the centipede *S. maritima* as the outgroup (Fig. 1a). Strong bootstrap support was obtained for spider, mite and tick clades, forming a monophyletic group. *T. tridentatus* and *L. polyphemus* were grouped together, forming the Xiphosura clade. The comparative genomic analysis of the 14 species revealed 14,479 orthologous groups containing genes in at least two different species, among which 1993 shared groups were commonly distributed in all sampled species, with 111 single-copy orthologues (Fig. 1b). The single-copy genes enriched for KEGG pathways such as ribosome, oxidative phosphorylation, proteasome, metabolic pathways, and carbon metabolism. Additionally, *T. tridentatus* and *L. polyphemus* had the most orthologues shared among these two species (2720 (22.2%) and 2648 (21.5%)). Pathway enrichment of these genes showed significant enrichment (*p*-value < 0.01) for neuroactive ligand-receptor interaction, FoxO signalling pathway and AGE-RAGE signalling pathway in diabetic complications. The latter two KEGG pathways include the important JAK-STAT signalling pathway genes related to innate immunity in arthropods. With respect to species-specific genes, 1124 genes were unique to *T. tridentatus*. *C. sculpturatus* had the most (7328) expanded species-unique genes, followed by 6247 *N. clavipes*-specific gene families. In contrast, only 161 genes were unique to *T. mercedesae*. The numbers of species-specific genes in *T. tridentatus* and *L. polyphemus* were in between, with 1124 and 857, respectively. Nevertheless, considering the fragmentation of the draft genome, there may be unidentified coding genes in the analysed genomes. The species-specific genes described here only refer to the results based on the draft genomes.

**Fig. 2:**
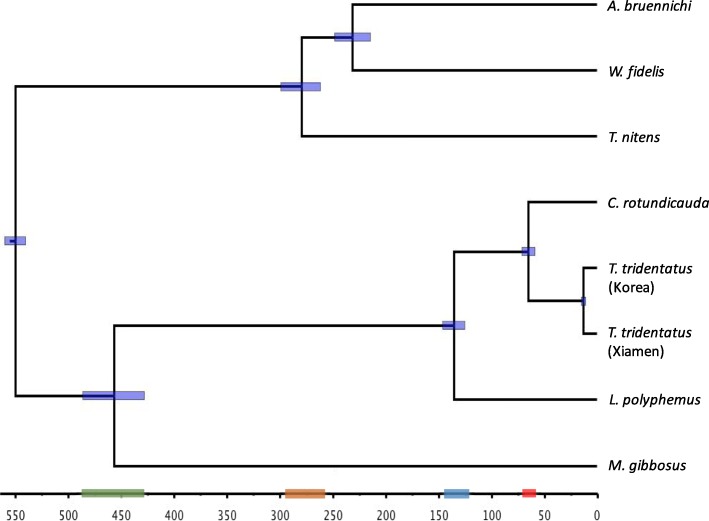
Bayesian maximum-clade-credibility tree based on the concatenated mitochondrial coding genes dataset in BEAST 2.5.1 with a strict clock, showing the estimated divergence time of Chelicerata species. Node shows the mean estimated divergence times in million years ago (MYA). Purple bars indicate 95% confidence levels. On the time axis, the green bar shows the divergence time for split of the scorpion from horseshoe crabs; the brown bar shows the inner split time of the three spiders; the blue bar shows the origin of the the last common ancestor of Asian horseshoe crabs (including *T. tridentatus*) and *L. polyphemus*; the red bar shows the inner split of *C. rotundicauda* and *T. tridentatus*

The divergence time estimate results for the 7 Chelicerata species showed that the last common ancestor of Asian horseshoe crabs (including *T. tridentatus*) and *L. polyphemus* was dated to 130 Mya (121–141) and that the split of the Asian horseshoe crabs *T. tridentatus* and *C. rotundicauda* was dated to 63 Mya (Fig. 2), while the internal split of *T. tridentatus* from southern coastal China to the Korean Peninsula was dated to 12 Mya. Both the species tree and time tree suggested that horseshoe crabs are closely related to scorpions and that the split of scorpions from horseshoe crabs was dated to 440 Mya (412–468).

### Two Hox gene clusters

Hox genes, which are a highly conserved subclass of homeobox super-class genes that have been extensively investigated, are usually distributed in clusters [34, 35]. Analysis of the Hox gene family showed that the *T. tridentatus* assembly contained 46 Hox genes, while 43 Hox genes were identified in *L. polyphemus* (Additional file 1: Table S1). This is the most complete set of Hox genes we obtained based on homeobox domains from these two horseshoe crab assemblies. We found that most Hox genes had at least two representatives in both genomes, which was consistent with a previous whole-genome duplication study in horseshoe crabs [36].

We further examined the positions of the identified Hox genes in the two genomes and found two clusters of adjacently distributed Hox1 and Hox4 in the *T. tridentatus* assembly. In *L. polyphemus*, there was one Hox cluster of adjacent Hox1 and Hox4 genes and one additional Hox1, Hox2 and Hox3 cluster. Other clusters, such as adjacent Hox2 and Hox3 clusters and longer clusters of Hox4, Hox7, Ubx, AbdA and AbdB genes found in the two assemblies, could probably be connected to the two clusters mentioned above. Based on the Hox gene positions in the assemblies, our analysis is consistent with a previous study and suggests that there are possibly two Hox gene clusters present in horseshoe crabs if Hox genes are linearly arranged in clusters along the anterior-posterior axis similar to the ancestral arthropod *Drosophila* [37].

### Expansion of crucial gene families of the innate immune signalling pathways in *T. tridentatus* and *L. polyphemus*

**Fig. 3:**
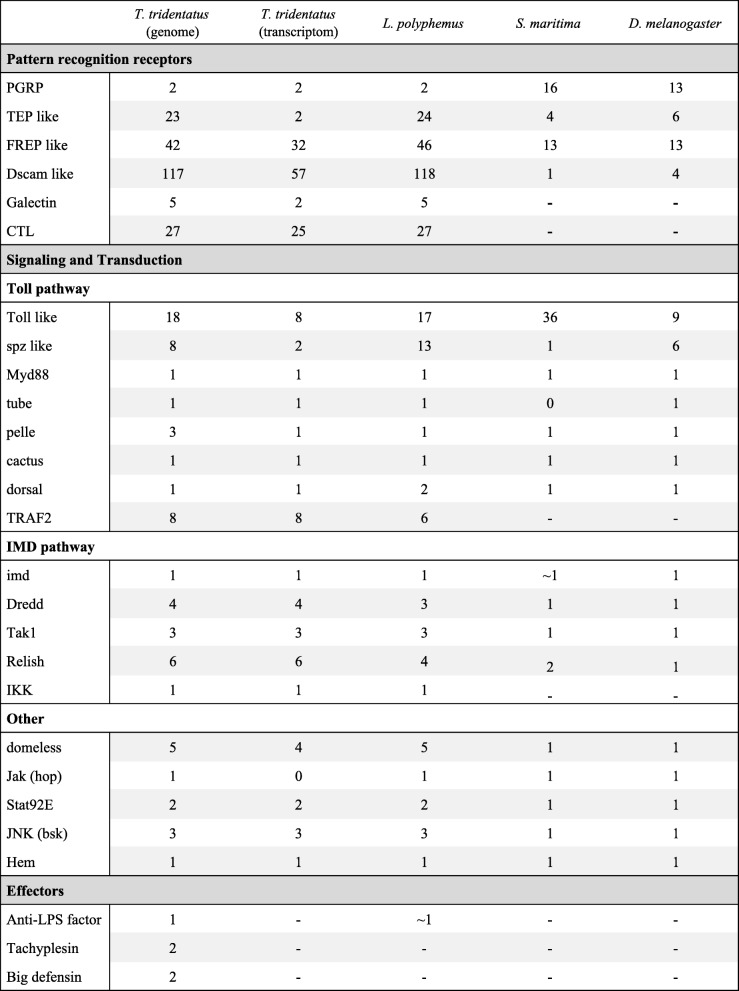
Presence of immune related gene families in *T. tridentatus* and *L. polyphemus*. Counts of immune related genes are shown for *T. tridentatus*, *L. polyphemus*, *S. maritima* [38] and *D. melanogaster*. The gene number counts according to results of BLASTP search in NR database and InterPro protein domain search from the genome of *T. tridentatus* and *L. polyphemus* and the transcriptome of *T. tridentatus*. Abbreviations: PGRP, peptidoglycan recognition protein; TEP, thioester-containing protein; FREP, fibrinogen-related protein; CTL, C-type lectin

Immune-related genes can be broadly classified into pattern recognition receptors (PRRs), signaling transduction pathways and effectors. We manually searched the *T. tridentatus* and *L. polyphemus* genomes and *T. tridentatus* transcriptome for homologues of essential immune-related genes. PRRs in *T. tridentatus* and *L. polyphemus* show large amounts of expansion, and key genes in the signal transduction pathways also exhibit a certain degree of expansion (Fig. 3). We examined six PRR families in *T. tridentatus* and *L. polyphemus*, which included the peptidoglycan recognition proteins (PGRPs), thioester-containing proteins (TEPs), fibrinogen-related proteins (FREPs), down syndrome cell adhesion molecules (Dscams), galectins and C-type lectins (CTLs). The results revealed 42 FREPs and 117 Dscams in *T. tridentatus* that were extensively present in both horseshoe crab genomes with functional domains.

Recognition of PAMPs by PRRs triggers signal transduction pathways through transcriptional activation. All known gene family components that play important roles in innate immune signal transduction in arthropods (such as the Toll, IMD, JAK-STAT, and JNK pathways) [39–41] are present in the genomes of *T. tridentatus* and *L. polyphemus*. We found that IMD and JAK-STAT pathway genes in *T. tridentatus* and *L. polyphemus* exhibited a certain degree of expansion. The orthologue analysis for shared genes in horseshoe crabs with their close evolutionary related species showed that horseshoe crabs have the most unique (more than twenty percent) uniquely shared gene orthologues, including the abovementioned expanded gene families.

**Fig. 4:**
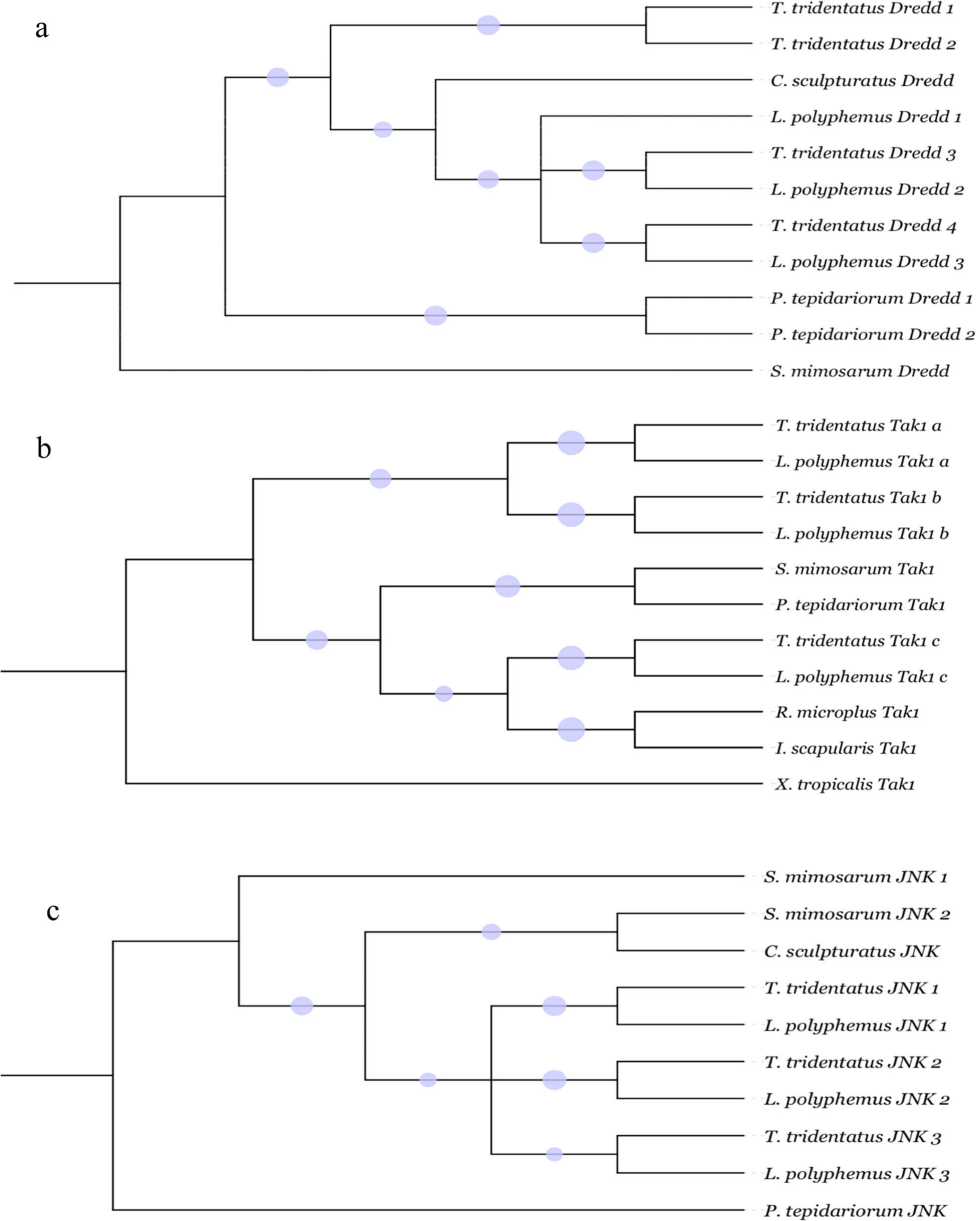
Phylogenetic analysis of immune signaling pathway related genes. **a** Phylogeny of Dredd genes involved in IMD signaling pathways among multiple Chelicerata species. **b** Phylogeny of Tak1 genes involved in IMD signaling pathways among multiple Chelicerata species. **c** Phylogeny of JNK genes involved in JNK signaling pathways among multiple Chelicerata species. The two Neighbor-Joining and one Maximum Likelihood trees were constructed using MEGA with 1000 bootstrap, and rooted with *S. mimosarum* for Dredds, *X*. *tropicalis* for Tak1 and *P. tepidariorum* for JNKs

Regarding the IMD signalling pathway, imd and IKK exit as a single gene, and we discovered multiple copies of genes encoding death-related ced-3/Nedd2-like proteins (Dredds), MAPKKK transforming growth factor - β (TGFβ) - activated kinase 1 (Tak1) and Relish proteins within *T. tridentatus* and *L. polyphemus*. For Dredds, the phylogeny tree shows one branch including 7 corresponding genes identified in the two horseshoe crabs and 1 gene in *C. sculpturatus*. Another branch encompasses 2 genes in *P. tepidariorum* (Fig. 4a). The Dredds are required for Tak1 activation. For Tak1, one branch consisting of two gene copies in *T. tridentatus* and *L. polyphemus* suggested gene expansion (Fig. 4b). Moreover, main components of the JAK-STAT signalling pathway, including the receptor Domeless and the Janus Kinase and STAT transcription factor, were identified in both *T. tridentatus* and *L. polyphemus*, indicating that the JAK-STAT pathway has remained intact in horseshoe crabs. Two STAT homologue candidates were identified in the *T. tridentatus* genome with the typical functional domains, including a DNA binding domain and an SH2 domain which are conserved compared to those reported in insects and shrimps [42]. Plausible homologs of major components of the JNK signalling were also identified in both *T. tridentatus* and *L. polyphemus*. Phylogenetic analysis of JNKs showed that there were three branches consisting of a pair of corresponding genes identified in the *T. tridentatus* and *L. polyphemus* genomes and one branch formed by a pair of genes in *C. sculpturatus* and *S. mimosarum* (Fig. 4c).

### Antimicrobial peptide diversity in *T. tridentatus*

A hallmark of the *T. tridentatus* host defence system is the production of antimicrobial peptides, which act as innate immune effectors [43]. We searched the *T. tridentatus* genome for antimicrobial peptide genes and identified most of the antibacterial peptides that have been reported, including one anti-LPS, two tachyplesin and two big defensin peptides (Fig. 3).

The anti-LPS gene found in the *T. tridentatus* genome contains an antimicrobial peptide (AMP) region between G23 to R83 with two conserved cysteine residues as well as a hydrophobic NH^2^-terminal and cationic residues clustered in its disulphide loop, which are supposed to act as an affinity site in combination with LPS [44, 45]. The tachyplesin family includes constitutively expressed cationic peptides comprised of 17–18 amino acids that strongly inhibit the growth of both Gram-negative and -positive bacteria, including pathogenic microorganisms from marine bivalves such as *Bonamia ostreae*, *Perkinsus marinus* and Vibrio P1, and can also have strong inhibitory effects on the growth of fungi [46, 47]. In this study, we identified two tachyplesin precursors in *T. tridentatus*, each of which consists of 77 amino acids encompassing a putative signal peptide sequence, a mature tachyplesin peptide sequence, a C-terminal arginine followed by the amidation signal residues Gly-Lys-Arg and a 22-aa peptide in the C-terminal portion [47]. In addition to this, two big defensin protein precursors are also present in the *T. tridentatus* genome, one of which is 118 amino acids in length and contains a hydrophobic N-terminal half and a cationic C-terminal half, which may be closely related to its biological activity for broad antimicrobial properties [48].

### Intact coagulation cascades in *T. tridentatus*

Serine protease-dependent rapid coagulation in horseshoe crabs has been shown to play a key role upon the activation of immune pathways in response to pathogen detection [49]. We found that *T. tridentatus* and *L. polyphemus* have all the coagulation-related genes while other related species lack a part of the coagulation pathway (Table 2), indicating a wider diversity of coagulation factors and a relatively intact coagulation cascade present in horseshoe crabs. Factor G, a heterodimer that is specifically activated by the fungal cell wall component 1,3-β-D-glucan, is a special serine protease precursor that provides another starting point for the clotting reaction [50, 51]. We identified 4 factor G sequences in our *T. tridentatus* genome and transcriptome assembly, including genes encoding the alpha and beta subunits, respectively. However, we failed to identify any clotting factor G homologues in other Chelicerata species.

**Table 2:** Coagulation Cascade genes in 2 horseshoe crabs, 1 scorpione, and 3 spiders

	Horseshoe Crabs		Scorpiones	Spiders		
Species	*T. tridentatus*	*L. polyphemus*	*C. sculpturatus*	*N. clavipes*	*S. mimosarum*	*P. tepidariorum*
Factor C	2	4	5	4	6	4
Factor B	10	12	8	4	7	7
Factor G	4	1^a^	0	0	0	0
Proclotting Enzyme	9	11	7	5	5	5
Coagulogen	6	6	0	0	0	0
Total	30	33	20	13	18	16

^a^Identified in previous study

## Discussion

The draft genome of *T. tridentatus* can provide the Chelicerata clade another high-quality publicly available sequence, and would provide an important source for eliminating the uncertainty associated with the evolution of Chelicerata. To date, two papers describing the *T. tridentatus* genome have been published, revealing 2.16 Gb and 1.94 Gb *T. tridentatus* genomes, providing valuable genomic and transcriptomic resources for future studies to exploit horseshoe crabs [52, 53]. Using a parallel experiment, the assembly size in this study was between the two previous *T. tridentatus* assemblies. Besides, the number of protein-coding genes predicted in *T. tridentatus* genome was lower that that from the other two published *T. tridentatus* genomes (34,966 and 25,252) but higher than that from *L. polyphemus* (23,287) [18–20]. Considering that previous phylogenetic studies only used transcriptomic data with multiple representations of one gene or obtained low bootstrap support for Arachnida, our phylogenetic tree using 111 single-copy orthologous groups of 13 Chelicerata species and 1 outgroup does not support the hypothesis that Euchelicerata are composed of two parallel groups, the Xiphosura and the Arachnida. Even so, the relatively wider species sampling range and more comprehensive information of this study would be helpful to explore the Chelicerata taxa. We further investigated the divergence time using mitochondrial coding sequences from 7 Chelicerata species, and our analyses suggest that the diversification of the *Limulidae* and *T. tridentatus* lineages was congruent approximately 121–141 Mya, and the lineages of the two Asian horseshoe crabs *T. tridentatus* and *C. rotundicauda* was also congruent approximately 57–69 Mya. According to the continental drift theory, before the Triassic Period, virtually all continents were joined to form the supercontinent Pangea, with the breakup of Pangea commencing in the Triassic Period [54]. Approximately 170–120 million years ago (MYA), Pangea broke up into the following two supercontinents: Laurasia and Gondwana [55]. The subsequent lineage divergence within reptiles [56], amphibians [57, 58], mammals [59] and even plants [60] matches the separation and fragmentation of Laurasia and Gondwana. Laurasia fragmented during the mid-Mesozoic Era [61], but until late-Cretaceous Period, the Eurasian and North American plates were still joined together [62]. The ancestor of horseshoe crabs (or their progenitor species) likely originated in the Mesozoic waters of Europe [63, 64]. After the final breakup of the Eurasian and North American plates, the European land mass formed as the shallow seas disappeared and the ancestors of the horseshoe crab migrated. One group migrated to the west along the east coast of North America from Maine to south Florida and from the Gulf of Mexico to the Yucatan Peninsula and evolved into the Atlantic species *L. polyphemus*. The second group migrated to the east through the Tethys, is found along Asia from Japan to India, and evolved into *T. tridentatus*, *T. gigas*, and *C. rotundicauda*. There is evidence showing that the India–Asia collision was underway in northern Pakistan ca. 56–55 Mya [65]. The diversity of the Asian horseshoe crabs *T. tridentatus* and *C. rotundicauda* may be related to the India–Asia collision.

Hox genes have been well studied to play essential roles in determining the anterior-posterior axis as well as organogenesis during embryonic development [24, 25]. Research focused on Hox genes suggests the duplication of clusters from one common ancestor [26]. Examination of Hox cluster genes with detailed scaffold positions in the *T. tridentatus* and *L. polyphemus* genomes in this study revealed two Hox gene clusters, suggesting that one round of whole-genome duplication occurred in *T. tridentatus* and *L. polyphemus*. Considering that draft genome data was utilized, the identification and scaffold positions in the Hox genes analysis in both horseshoe crab genomes probably provide a minimum estimate. More intact Hox clusters may be recovered with more complete assemblies; thus, we can assume that at least one round of whole-genome duplication occurred in both *T. tridentatus* and *L. polyphemus*.

Horseshoe crabs are an independent ancient group with distributions in Southeast Asia and North America. There must be inherent reasons for their survival against complicated marine environmental changes. Some clues could be found from the comparison of the previously determined *L. polyphemus* genomes and our presented *T. tridentatus* genome. Among them, the immune system undoubtedly provides an important guarantee. We searched the two horseshoe crab genomes to investigate the molecular basis of immune signalling pathways, which affect the specificity of foreign pathogen recognition and performance for releasing immune response substances. Genes that are not found in the *T. tridentatus* genome were supplemented by transcriptome data to restore as accurate of numbers as possible. Although the draft genome is incomplete, the gene count errors should be small enough for the predicted genes according to the BUSCO evaluation results of 98.7% conserved homologous proteins. From the results we have obtained, the two horseshoe crabs showed high sequence homology for most immune signaling-related gene families, which have been studied previously in other arthropods. Homologs of the FREP and Dscam families play an important role, and pattern recognition receptors with their corresponding functional domains were extensively present in the two horseshoe crab genomes. The Dscam family in arthropods has evolved to recognize a variety of pathogens, and this is supported by the abundant receptor diversity due to alternative splicing of hypervariable regions [27–30]. The abundantly expressed PRRs in the *T. tridentatus* transcriptome suggest an effective ability to recognize a broad range of pathogens, which may be inducted to cope with a great diversity of invading microorganisms in the marine environment. Pathogen recognition would further lead to the activation of signal transduction and the amplification of immune responses, thus producing immune factors that are resistant to microbial activity. Several gene family members in the IMD and JAK-STAT signalling transduction pathway exhibit a certain degree of expansion in *T. tridentatus* and *L. polyphemus*. Research in Drosophila has shown that the IMD pathway preferentially recognizes the peptidoglycan molecules present on the surface of Gram-negative bacteria through peptidoglycan recognition receptors (PGRPs), leading to the generation of specific AMPs. The JAK-STAT pathway in arthropods, which is analogous to a cytokine-signalling pathway in mammals, has been implicated as having defence abilities against viral, bacterial or protozoan pathogens [31–33]. The expanded PRRs and signalling pathways might predict that the innate immunity of *T. tridentatus* has strong signal reception and lineage-specific signal transduction abilities. The interactions among the components in *T. tridentatus* should be paid more attention.

The generation of AMPs is an important aspect of immune responses in horseshoe crabs. The transcription of specific AMPs, which act as important innate immune effectors, are activated through the Toll, IMD, JAK-STAT or JNK pathway based on the recognition of bacteria, fungi, viruses or parasites. We identified most of the antimicrobial peptides isolated in previous studies in *T. tridentatus* assembly, including anti-LPS, tachyplesin, and big defensin peptides. However, in the *L. polyphemus* genome, we failed to find any of the tachyplesin family genes, probably because these antimicrobial peptides are usually shorter and have a high degree of species specificity. There is increasing evidence that AMPs in horseshoe crabs not only possess broad-spectrum antimicrobial capabilities but also have a strong resistibility to enveloped viruses, parasites and tumour cells [66–69]. Moreover, antimicrobial peptide counterparts in horseshoe crab haemolymph have also been identified in other evolutionarily conserved animal population representatives [70–72]. Thus, more work needs to be undertaken to study the specific function of the AMPs in *T. tridentatus*.

Serine protease-dependent haemolymph coagulation is a major component of the innate immune system in horseshoe crabs. Activation of the horseshoe crab coagulation cascade consists of four coagulation factors including factor C, factor B, factor G and proclotting enzyme [73, 74]. Factor C and factor G are two serine protease zymogens that act as biosensors and can be activated by LPS or (1 → 3)-β-D-glucan, which are major components of the cell walls of Gram-negative bacteria and fungi, respectively. We found relatively high number of genes for all four coagulation factors in the *T. tridentatus* assembly, indicating a complete coagulation cascade in horseshoe crabs. Other Chelicerata species can lack factor G and coagulogen. This might be because of a limited annotation of the draft genome assemblies or there might be other special biosensors involved in their antifungal recognition process*.* Although the more direct internal cause for the long-term evolutionary conservation and success of horseshoe crabs with abilities to maintain morphological stability in a fluctuating marine environment may be the combination of a wide feeding spectrum, substantial saline tolerance and insensitivity to temperature by horseshoe crabs [75, 76], there is a presumption that the haemolymph of horseshoe crabs may improve their adaptive strategies and increase their population survival rate [77, 78].

## Conclusions

Our draft assembly of *T. tridentatus* was sequenced into a 2.06 Gb of genome consisting of 143,932 scaffolds with an N50 scaffold size of 165 kb. In total, 24,222 protein-coding genes were conservatively predicted as present in the *T. tridentatus* genome, revealing 98.7% completeness with 1052 BUSCOs.

Further analysis of the *T. tridentatus* genome included phylogeny analysis and divergence time dating using newly published Chelicerata species, as well as selective analysis of Hox genes, innate immunity-related genes, coagulation factors and antimicrobial peptides. We found that the two horseshoe crabs *T. tridentatus* and *L. polyphemus* had the most orthologues shared among two species, and were enriched for the immune-related JAK-STAT signalling pathway. Furthermore, two clusters of Hox genes were predicted in both assemblies, suggesting that at least one round of whole-genome duplication occurred in both *T. tridentatus* and *L. polyphemus*. The innate immunity gene investigation showed that conserved pattern recognition receptor gene families and several signal transduction pathway genes involved in IMD and JAK-STAT exhibit a certain degree of expansion in both horseshoe crab genomes. Moreover, all of the antibacterial peptides reported in previous studies, as well as their effective antimicrobial sites, have also been identified in the *T. tridentatus* genome. Beyond that, according to our analysis, intact coagulation cascade-related genes were identified in the *T. tridentatus* genome, with high numbers of total gene counts. Apart from the abovementioned results, there may remain other aspects of horseshoe crabs that may be greatly conducive to their adaptive advantages in marine environments through their long evolutionary history that need to be studied further and established in future studies.

## Methods

### *T. tridentatus* specimens and DNA extraction

*T. tridentatus* is endangered according to the Red List (https://www.iucnredlist.org/species/21309/149768986). The use of the *T. tridentatus* species collected in this study was to separate 0.1 g leg skeleton muscle tissue for DNA extraction. This study was reviewed and approved by the Animal Care and Use Committee of Minjiang University. All the required collection and permits had been obtained before any work was started. Only one *T. tridentatus* was purchased in the aquatic market in Xiamen, Fujian Province, China and was released after 0.1 g leg muscle collection. Total genomic DNA was extracted using an E.Z.N.A.® Insect DNA Extraction Kit (Omega Bio-Tek, Inc., Norcross, GA, USA) at the State Key Laboratory of Genetic Engineering in Fudan University, Shanghai, China. The genomic research about *T. tridentatus* covers the evolution history of this endangered species, and may reveal genomic characteristics that limit their survival, which could provide a basis for the species protection.

### Genome sequencing and assembly

The Illumina TruSeq Nano DNA Library Prep Kit (Illumina, San Diego, CA) was used to construct the 400-bp and 800-bp paired-end sequencing libraries. Long mate-pair libraries featuring 3-kb inserts were prepared using a CHGC kit (CHGC, China). The Nextera MP Sample Prep Kit (Illumina, San Diego, CA) was used to build long mate-pair libraries with 8- and 12-kb insert sizes. Whole-genome sequencing was performed on the Illumina HiSeq 2500 and HiSeq X Ten platforms generating 250-bp and 150-bp paired-end reads, respectively. Five libraries of nominal insert sizes, including 400 bp, 800 bp, 3- kb, 8- kb and 12- kb, were sequenced at expected genome coverages of 60×, 30×, 7×, 7× and 20×, respectively. The generated clean reads from the 400 bp, 800 bp and 3- kb insert size libraries were used for the estimation of the genome size via JELLYFISH v1.116 [79] with a k-mer length of 17. The genome size of *T. tridentatus* was estimated using online scripts (https://github.com/josephryan/estimate_genome_size.pl) from a k-mer distribution generated by jellyfish. Contigs were assembled using Velvet [80] by cutting the short reads into k-mers and establishing the de Bruijn table to correct and complete the contigs, which were further scaffolded and gap-filled using SSPACE-STANDARD [81] with long mate-pair reads (8- and 12-kb).

### Automated annotation

*T. tridentatus* genome assembly automatic gene prediction was established using AUGUSTUS (Version 3.3) [82]. The predicted genes were annotated by comparing to the NCBI non-redundant database (NR), Kyoto Encyclopedia of Genes and Genomes (KEGG) database [16] and InterPro database [17] with an E-value threshold of 10^− 5^. The three annotation results were combined as the annotation of the predicted genes. The benchmarking sets of universal single-copy orthologues (BUSCOs) [21] were used to assess the completeness of the predicted genes with 1066 Arthropoda datasets. The repeat contents of *T. tridentatus* and *L. polyphemus* were first analysed using RepeatMasker (version open-4.0.5) [18] with merostomata as the query species and running with rmBLASTn (version 2.2.27+) [83] and RepBase (version 20,140,131) [84]. RepeatModeler (version open-1.0.11) [19] was used to build the repeat database, which was further masked by RepeatMasker.

### Transcriptome analysis

RNA-seq raw data obtained from the embryonic sample at Stage 21 (the hatch-out stage) of *T. tridentatus* were downloaded from the NCBI SRA database (accession number SRX330201) [20]. De novo assembly of the transcriptome was performed with Trinity [85] with default parameters. Protein-coding sequences and the longest transcript ORFs were predicted via Transdecoder [85].

### Orthology and phylogeny analysis

The published genome assembly, coding sequences and protein sequences for the Atlantic horseshoe crab *L. polyphemus* submitted by Washington University were downloaded from NCBI with RefSeq ID 2304488, accession GCF_000517525.1. The non-redundant protein sequences in *L. polyphemus* were selected by sorting the protein scaffold positions and filtering out overlapped proteins. Protein sequences of other 11 Chelicerata species, including three Araneae (*Nephila clavipes*, *Parasteatoda tepidariorum*, and *Stegodyphus mimosarum)*, seven Acari (*Varroa jacobsoni*, *Tropilaelaps mercedesae*, *Sarcoptes scabiei*, *Metaseiulus occidentalis*, *Tetranychus urticae*, *Varroa destructor*, and *Ixodes scapularis*), and one scorpion (*Centruroides sculpturatus*) were downloaded from the GenBank database. Additional outgroup protein sequences from the centipede *Strigamia maritima* were downloaded from the UniProt database. OrthoMCL [23] was used to perform orthologous gene family clustering and provide calculation results for the number of genes in each orthologous group. Selected single copy orthologues were subjected to multiple sequence alignments using ClustalW v2.0.12 [86] and Maximum Likelihood phylogenetic trees were built using the PROTGAMMAWAG model with 1000 bootstraps implemented in RAxML v8.2.12 [87], with centipede as an out-group. The KOBAS 3.0 web server [88–90] was used for the functional gene set enrichment of shared and species-specific genes from *L. polyphemus* and *T. tridentatus* and the single-copy gene set for the 13 Chelicerata species and centipede with the KEGG database [16].

### Divergence time estimates

Thirteen intact mitochondrial coding sequences of 7 species were downloaded from the NCBI gene database and used to build the time tree containing three horseshoe crabs (*Tachypleus tridentatus*, *Carcinoscorpius rotundicauda* and *Limulus polyphemus*), one scorpion (*Mesobuthus gibbosus*) and three Araneae (*Argiope bruennichi*, *Wadicosa fidelis* and *Tetragnatha nitens*). The 13 mitochondrial coding sequences for *T. tridentatus* collected in Korea were used to query for identical genes in our *T. tridentatus* genome and transcriptome assembly using blastn (blast, Basic Local Alignment Search Tool). Alignment of sequences with identities of at least 97% was considered to be the counterparts of mitochondrial coding sequences in *T. tridentatus* identified in this study. Multiple sequence alignments of concatenated mitochondrial coding sequences of 7 species were processed using ClustalW v2.0.12 [86]. BEAUti v2.5.1 [91–93] was used to generate the BEAST input XML files, after which the tree was dated using BEAST v.2.5.1. The Hasegawa-Kishino-Yano (HKY) substitution model with empirical frequencies and a strict clock model were used. Fossil information for all Chelicerata species were used to calibrate the tree with a normal distribution of 530 Mya and standard deviation 5 Mya [94]. A chain length of 6,000,000 generations was run when sampling every 1000 generations. Software Tracer was used to analyse the BEAST log file output. TreeAnnotator [95] and FigTree were used for tree production and tree visualization, respectively.

### Identification of Hox genes, immune pathway genes and coagulation factors

Protein sequences of Hox genes, essential immune signalling-related genes and coagulation factors of species closely related to horseshoe crabs were downloaded from the NCBI protein database and used as query sequences. Blastp was then used with an e-value of 10^− 15^ to search for homologues using the *T. tridentatus* and *L. polyphemus* genomes as databases. Tblastn was used with an e-value of 10^− 15^ to search for corresponding transcripts in the *T. tridentatus* transcriptome. Transcripts found in the transcriptome were further compared to the genome of *T. tridentatus* by blastn with an e-value of 10^− 5^ to complement identified genes and to reduce the data omission in the genome. Putative genes were selected based on positive scores and alignment length percentages defined by dividing the alignment length by query length, and then filtrated according to their annotation in the NCBI non-redundant database (NR) and InterPro database [17].

### Dredd, Tak1 and JNK gene phylogenetic analysis

MEGA (version 7.0) [96] was used to construct two neighbour-joining trees and a maximum likelihood tree with 1000 bootstraps using putative protein sequences as the default parameters. Domains from all the selected genes annotated as IPR001309, IPR001932 and IPR000719 in the InterPro database were used for multiple alignment. Dredd from *Stegodyphus mimosarum*, Tak1 from *Xenopus tropicalis* and JNK from *Parasteatoda tepidariorum* were chosen as outgroups for the above phylogenetic trees.

### Identification of putative antimicrobial peptides

Because antimicrobial peptides (AMPs) are commonly short in length, display wide variety and have large differences in their structures and functions within species, tblastn was used to identify potential AMPs using the *T. tridentatus* and *L. polyphemus* genome assemblies as databases. Identified putative AMPs were further used as query sequences to search for corresponding transcripts. The prediction of ORFs was performed using NCBI ORF finder (https://www.ncbi.nlm.nih.gov/orffinder/) and the annotation of those ORFs was performed by blastp with the NR database.

## Supplementary information

**Additional file1: Table S1.** Comparison of homeobox ANTP class genes between *T. tridentatus* and *L. polyphemus* genome.

## Data Availability

All data are available from the following databases as described. The raw sequences for *T. tridentatus* have been deposited in the NCBI SRA: BioProject ID PRJNA510236, BioSample ID SAMN10600682, accession SRX6412182 - SRX6412188. The whole genome assembly has been deposited at GenBank under the BioProject ID PRJNA510236, accession QCWK00000000. The version described in this paper is version QCWK01000000. RNA-seq raw data obtained from the embryonic sample at Stage 21 (the hatch-out stage) of *T. tridentatus* were downloaded from the NCBI SRA database (accession number SRX330201). The published genome assembly, coding sequences and protein sequences for the Atlantic horseshoe crab *L. polyphemus* submitted by Washington University were downloaded from NCBI with RefSeq ID 2304488, accession GCF_000517525.1. Protein sequences of three Araneae (*Nephila clavipes*, *Parasteatoda tepidariorum*, and *Stegodyphus mimosarum*), seven Acari (*Varroa jacobsoni*, *Tropilaelaps mercedesae*, *Sarcoptes scabiei*, *Metaseiulus occidentalis*, *Tetranychus urticae*, *Varroa destructor*, and *Ixodes scapularis*), and one scorpion (*Centruroides sculpturatus*) were downloaded from NCBI Genbank with following accession numbers: GCA_002102615.1, GCF_000365465.2, GCA_000611955.2, GCA_002532875.1, GCA_002081605.1, GCA_000828355.1, GCA_000255335.1, GCA_000239435.1, GCA_002443255.1, GCA_000208615.1 and GCA_000671375.2. 15,047 protein sequences from the centipede *Strigamia maritima* were downloaded from UniProt (https://www.uniprot.org/uniprot/?query=Strigamia+maritima&sort=score). Thirteen intact mitochondrial coding sequences of 7 species were downloaded from the NCBI gene database with following accession numbers: 7768785–7768797 for *Tachypleus tridentatus*, 14049643–14049655 for *Carcinoscorpius rotundicauda*, 803775–803787 for *Limulus polyphemus*, 3183285–3183286, 3183289–3183291, 3183293, 3183295, 3183297–3183298, 3183300, 3183305, 3183313, 3183315 for *Mesobuthus gibbosus*, 19591985–19591997 for *Argiope bruennichi*, 22834025–22834037 for *Wadicosa fidelis* and 26047144, 26047146–26047148, 26047151, 26047156, 26047158, 26047160, 26047167–26047170, 26047172 for *Tetragnatha nitens.*

## Abbreviations

AbdA: Abdominal-A
AbdB: Abdominal-B
AMP: Antimicrobial Peptide
blast: Basic Local Alignment Search Tool
BUSCO: Benchmarking Universal Single-Copy Orthologs
Dscams: Down Syndrome Cell Adhesion Molecules
FoxO: The Forkhead Box O
FREPs: Fibrinogen-Related Proteins
Hox genes: Homeotic Genes
IMD: Immune deficiency
JAK: Janus kinase
JNK: c-Jun N-terminal Kinase
KEGG: Kyoto Encyclopedia of Genes and Genomes
LINEs: Long Interspersed Elements
LPS: Lipopolysaccharide
LTR: Long Terminal Repeat
ORF: Open Reading Frame
PAMPs: Pathogen-Associated Molecular Patterns
PRRs: Pattern Recognition Receptors
RAGE: Receptor for Advanced Glycation Endproducts
STAT: Signal Transducer and Activator of Transcription Protein
Ubx: Ultrabithorax
