# Supplementary material for: The draft genome of horseshoe crab Tachypleus tridentatus reveals its evolutionary scenario and well-developed innate immunity
Source: BMC Genomics. 2020 Feb 10;21:137. doi: 10.1186/s12864-020-6488-1 (PMC7011531; doi:10.1186/s12864-020-6488-1)
Supplement: Supplementary file 1 — Additional file1: Table S1. Comparison of homeobox ANTP class genes between T. tridentatus and L. polyphemus genome. [file 12864_2020_6488_MOESM1_ESM.doc]

**Table S1. Comparison of homeobox ANTP class genes between *T. tridentatus* and *L. polyphemus* genome.**

|  | *Tachypleus tridentatus* | *Limulus polyphemus* |
| --- | --- | --- |
| Lab/Hox1 | 3 | 3 |
| Pb/Hox2 | 5 | 3 |
| Zen/Hox3 | 2 | 3 |
| Dfd/Hox4 | 7 | 7 |
| Scr/Hox5 | 3 | 4 |
| Antp/Hox6-8 | 4 | 2 |
| Ftz/Hox6-8 | 2 | 1 |
| Ubx/Hox6-8 | 4 | 3 |
| AbdA/Hox6-8 | 2 | 2 |
| AbdB/Hox9-13 | 5 | 5 |
| Cdx | 1 | 3 |
| Evx | 2 | 2 |
| Unpg/Gbx | 4 | 4 |
| Mnx | 2 | 1 |
